# Medication counselling practices amid COVID -19 pandemic and associated factors in drug retail outlets of Jimma town, Southwest Ethiopia: cross-sectional study

**DOI:** 10.4314/ahs.v22i4.62

**Published:** 2022-12

**Authors:** Bekele Boche, Kebenesa Angasu, Sintayehu Alemu, Mengist Awoke

**Affiliations:** 1 Department of Social and Administrative Pharmacy, School of Pharmacy, Faculty of Health Sciences, Institute of Health, Jimma University, Jimma, Oromia, Ethiopia; 2 Department of Midwifery, Faculty of Health Sciences, Institute of Health, Jimma University, Jimma, Oromia, Ethiopia; 3 Department of pharmaceutics, School of Pharmacy, Faculty of Health Sciences, Institute of Health, Jimma University, Jimma, Oromia, Ethiopia; 4 Department of clinical pharmacy, School of Pharmacy, Faculty of Health Sciences, Institute of Health Jimma, University, Jimma, Oromia, Ethiopia

**Keywords:** Counseling practice, COVID -19, drug retail outlet, Jimma

## Abstract

**Background:**

Patients' good understanding and awareness of drug information received at the drug retail outlet is paramount to gaining expected outcomes. In the COVID-19 pandemic, the routine counselling practice faced multifactorial challenges.

**Objectives:**

The study aimed to assess medication counseling practice and associated factors in drug retail outlets of Jimma town, southwest Ethiopia.

**Methods:**

A facility-based cross-sectional study was conducted using an interviewer administered questionnaire. The data were analysed by using SPSS version 23. A multivariable logistic regression model was used to identify factors associated with medication counselling practice.

**Results:**

A total of 180 pharmacy professionals were enrolled in the study, about half (51.1%) of the participants reported good medication counselling provision for their patients. In A multivariable logistic regression analysis, reduced pharmacist's level of communication (AOR=0.008; CI: 0.001–0.292; p= 0.009) and shortage of personal protective equipment (AOR=0.021; CI: 0.002–0.226; p=0.002) due Covid-19 were factors associated with poor medication counselling practice.

**Conclusion:**

Reduced level of communication and shortage of personal protective equipment due to Covid-19 were factors associated with poor medication counselling practice. In general, Jimma town health offices and Oromia Region Health bureau should struggle in association with other stakeholders to improve the identified bottleneck of pharmacist's counselling practice.

## Background

Drug use counseling is an interactive approach between the patient and the pharmacist, which focuses on the patient's requirements, beliefs, and perceptions about drug treatment 1. Pharmacists' medication counselling activities are the core part of pharmaceutical care services, which are currently getting the great focus of pharmacy professionals, and expanding rapidly to promote cost-effective patient treatment outcomes. Medication counseling during dispensing should be clear, accurate, understandable, and complete for the patients 2–4.

The detail of counseling information includes the importance of taking medicine, consequences of discontinuing or using it incorrectly, the dose of the medicine, route of taking, frequency of receiving, action should be taken if a patient misses a dose, duration of treatment, storage conditions, side effects and actions to be taken, lifestyle modification during treatment, drug-drug interaction, food-medicine interaction, re-checking whether the information is understood by the patient after advising. Patients' good understanding and awareness of drugs received at the dispensary unit is vital to gain expected medication outcomes 3,5,6.

The clinical and economic burden of inappropriate medication use is growing. Patients, without adequate information about their medications they cannot be effective collaborators in managing their care. So, patients and caregivers should perceive the counselling area as comfortable and confidential to help patients easily understand and remember their medication taking procedure 7,8.

However, pharmacists' insufficient knowledge on medication treatment, lack of updated drug information, high patient load, inappropriate counselling area, undervaluing the prominence of counseling, lack of commitment and poor communication were the factors that hampered medication counseling 9–11. In Ethiopian, blow 40% of pharmacists have provided an acceptable level of medication counselling, and this is due to many pharmacists viewing it as an extra activity. The outbreak of COVID-19 pandemic further complicates the problems (12,13). Community pharmacy setting is the widely accessible and major pharmaceutical care services for the community, which is largely affected by the COVID-19 pandemic. As a result, pharmacists are at risk of contracting the COVID-19 pandemic because of they are on the frontline of the fight. Many patients with mild symptoms without a confirmed diagnosis of COVID-19 will seek advice from the pharmacist; in addition, the daily interact with an expected of 500 consumers asking about cosmetics, supplementary food, minor illnesses, and filling of prescriptions. However, in a low-income country, implementing appropriate social distancing is very difficult to reduce pandemic transmission in a drug retail outlet due to the weak health care system setting. As consequence, pharmacists are prone to COVID-19 infection and these mainly reduce their effort to provide appropriate medication counselling practice 14–16.

Ethiopia faces a different challenge with the different waves of the Covid-19 epidemic as other low-income countries are also unable to stop their major casualties. According to a report by the Ethiopian Public Health Institute, a total of 372,334 total cases, 12,480 active cases and 6,804 died of coronavirus by the end of December, 2021. The Ethiopian Ministry of Health officially kicked off the COVID-19 vaccine at Eka Kotebe COVID-19 Hospital on March 13, 2021, by prioritizing health workers, the elderly and patients with the chronic disease over the age of 55. However, due to lack of sufficient vaccine doses, the country planed only to vaccinate 20% of its 110 million populations by the end of 2021. This implies the impact of covid-19 continues to impose its destructive effect on medication counselling practice across the country 17,18.

Even though some studies reveal that there were inadequate pharmacists' medications counselling practices globally and in Ethiopia, as far as our knowledge is concerned, during the Covid-19 pandemic there was no study conducted in the current study area other than some commentary on pharmacist's medication counseling practice. Jimma is the largest town in southwestern Ethiopia, where many pharmaceutical care services are provided, and this is why it's designated as the study area. Therefore, this study aimed to assess the pharmacist's medication counselling practice amid the COVID -19 pandemic in drug-retail outlets of Jimma town, southwest Ethiopia.

## Methods

### Study settings, design, and period

A facility-based cross-sectional study was undertaken in drug retail outlets of Jimma town, Southwest Ethiopia. The town is located 346 kilometers away from Addis Ababa, the capital city of Ethiopia. There was one medical center, one primary hospital, 4 health centers, 24 private pharmacies and 33 drug stores in the town. A total of 190 pharmacy professionals were working in drug retail outlets found in Jimma town. The study was conducted from June to September, 2021.

### Population

#### Study population

All the pharmacists working in drug retail outlets of Jimma town were the source population whereas, pharmacists who volunteered to participate in the study and found during the data collection period were the study population. All pharmacists actively working in Jimma town drug retail outlets were included in the assessment however, pharmacists with less than one month's experience at drug retail outlet, who have not volunteered to participate in the study and are not available during the data collection period were excluded from the study. Sample size and sampling technique Actively, 190 pharmacists were working in Jimma town drug retail outlets. Using the census sample method, we took all pharmacists who fulfilled eligibility criteria, 185

### Data collection procedures

Structured self-administered questionnaires were developed by reviewing different kinds of literature (9,11,16,19,20) which had four parts and were distributed face to face. Part I: sociodemographic characteristics (age, sex, level of education, nature of employment, monthly income, working sector, and year of experience) of study participants assessed with seven items. Part II: Patient's counselling activities of pharmacists and evaluated by 16 items on a 5-point Likert-type scale from (1, always to 5, never). Part III: the impact of COVID-19 on pharmacists' counseling practice and assessed by 9 items on a 4 Likert scale (1, not affected to 4, strongly affected). The average score is used to dichotomize the participant's responses into not affected (No) and affected (Yes). Part IV: potential barriers of pharmacist's medication counseling practice and evaluated by 9 items on the Likert scale type questioners (1, strongly agree to 5, strongly disagree). The average score was used to split the responses into two categories: factors that affect (Yes) and not affect (No) pharmacists' counseling practices.

### Outcome measurement

Patient's counseling activities evaluated by 16 items through a 5-point Likert scale. The average score of the scale was used as cut point to declare the counseling practice of pharmacists as adequate (above the average) and inadequate (below the average) in the drug retail outlet during dispensing.

### Data quality assurance

In order to maintain the quality of the data, the tool was pre-tested for 5% of people surveyed at Buno Badele town drug retail outlets found Oromia region, southwest Ethiopia. The necessary modifications were made including, wordings on the questionnaire before it was applied to the study population. In addition, the data was compiled, coded, and checked for internal consistency before analysis. A Cronbach alpha analysis was done for nine Likert scale items measuring the impact of COVID-19 pandemic on pharmacists' counselling practices and resulted in 0.844.

### Data analysis

The data were coded and entered into Epi data 4.2 then exported to the Statistical Package for Social Science (SPSS) 23 for analysis. Frequency and percentage of data were computed and the result is presented with a table and text as desired. The chi-square test was performed to test the adequacy of the cell before using the binary input regression. Bivariate logistic regression was carried out to investigate associations between the reported level of medication counseling and independent variables. Then, a backward, stepwise multivariate logistic regression [reported with adjusted odds ratios (AOR) with 95% confidence intervals] was performed including all explanatory variables with ap-value of < 0.25 on bivariate logistic regression to evaluate factors independently associated with the reported level of medication counseling. All p-values calculated were two-sided, and the statistical significance threshold was <0.05.

## Result

### Sociodemographic characteristics of the study participants

A total of 180 pharmacists participated in the study with the response rate of 97.3%. Of the participants, majority, 102 (56.7%), of them were males, 186(47.8%) participants were aged below 30 years, and 112(62.2%) were working in the public secto/span>r. Most of the respondents (72.8%) were degree and above. Regarding years of experience, more than half (58.3%) of the respondents had less than five years of experience (Table 1).

**Table 1:** Sociodemographic characteristics of the study participants in drug retail outlets of Jimma town, Southwest Ethiopia, 2021 (n=180)

Demographic profiles	Category	Frequency (%)
Gender	Male	102 (56.7)
	Female	78(43.3)

Age	<30 years	94 (52.2)
	≥30 years	186(47.8)

Educational status	Diploma	49(27.2)
	Degree and above	131(72.8)

Working sector	Public	112(62.2)
	Private	68(37.8)

Nature of employment	Full time	155 (86.1)
	Part time	25(13.1)

Monthly income	<5000 ETB	49(27.2)
	5000–7500 ETB	72(40)
	>75000 ETB	59(32.8)

Years of experience	<5	105 (58.3)
	5–10	60 (25)
	>10	15(8.3)

### Patient's counseling activities of pharmacists

When the activities of medication counseling were assessed, 147(81.7%), 120(66.7%), and 104(57.8%) of participants provided drug information constantly to their clients on the duration of therapy, drug name, and unit dose, and the frequency of administration, respectively. More than one-third (37.2%) of the respondents commonly delivered drug information on the side effects of the medication, while information on what to do if adverse reactions occur to their patient was reported by 68(37.8%) of them, only sometimes (Table 2).

**Table 2:** Pharmacists counseling practice in drug retail outlets of Jimma town, Southwest Ethiopia, 2021 (n=180)

Counseling activities	Always (%)	Often (%)	Sometimes (%)	Rarely (%)	Never (%)
Tell the drug name and unit dose	120(66.7)	15(8.3)	25(13.9)	14(7.8)	6(3.3)
Tell the frequency of administration	104(57.8)	50(27.8)	10(5.6)	7(3.9)	9(5)
Demonstrate the way of administration, if necessary	67(37.2)	34(18.9)	44(24.4)	14(7.8)	21(11.7)
Tell duration of therapy	147(81.7)	13(7.2)	4(2.2)	13(7.2)	3(1.7)
Tell drug–drug interaction	41(22.8)	53(29.4)	46(25.6)	38(21.1)	2(1.1)
Tell drug–food interaction	40(22.2)	58(32.2)	36(20)	40(22.2)	6(3.3)
Tell drug–drink interaction	57(31.7)	51(28.3)	29(16.1)	39(21.7)	4(2.2)
Counsel on major side effect	67(37.2)	50(27.8)	45(25)	15(8.3)	3(1.7)
Inform clients not to discontinue drugs without consulting healthcare provider	52(28.9)	42(23.3)	66(36.7)	11(6.1)	9(5)
Ask feedback from the clients	28(15.6)	26(14.4)	56(31.1)	56(31.1)	14(7.8)
Discuss lifestyle modifications	25(13.9)	38(21.1)	68(37.8)	36(20)	13(7.2)
Tell what to do if a patient misses a dose	45(25)	53(29.4)	44(24.4)	26(14.4)	12(6.7)
Provide information on what to do if adverse reactions happen	45(25)	46(25.6)	68(37.8)	10(5.6)	11(6.1)
Tell about storage conditions of the medicine	51(28.3)	37(20.6)	53(29.4)	27(15)	12(6.7)
Give written materials	41(22.8)	53(29.4)	42(23.3)	30(16.7)	14(7.8)

### Impact of COVID-19 on pharmacists counseling practice

In the current study finding, about half 46.7% of the respondents perceived that the level of communication with patients due to the Covid-19 pandemic was moderately affected medication counseling practice. Likewise, 62(34.4%), 50(27.8%), and 44(24.4%) of study participants believed that lack of appropriate counseling area to maintain physical distance protocol of the Covid-19, counseling practice time, and shortage of personal protective equipment imposed due to COVID19 pandemic were strongly affected medication counseling practice, respectively (Table 3).

**Table 3:** Impact of COVID-19 on pharmacists' medication counseling practice in drug retail outlets of Jimma town, Southwest Ethiopia, 2021 (n=180)

Activities impacted by covid- 19	Not affected	Slightly affected	Moderately Affected	Strongly Affected
Level of communication with patients	18(10)	27(15)	84(46.7)	51(28.3)
Your aim level to become involved in-patient counselling due to fear of covid 19 pandemic	21(11.7)	89(49.4)	29(16.1)	41(22.8)
Perception of counselling practice of pharmacy profession	23(12.8)	85(47.2)	24(13.3)	48(26.7)
The motivational level of counselling patient	30(16.7)	53(29.4)	37(20.6)	60(33.3)
Level of difficulty to counselling patient during the pandemic	40(22.2)	31(17.2)	58(32.2)	51(28.3)
Number of pharmacy professionals delivering dispensing in drug retail outlet due to covid -19	22(12.2)	89(49.4)	22(12.2	47(26.1)
Lack of counselling area to maintain physical distance protocol of COVID-19	13(7.2)	63(35)	42(23.3)	62(34.4)
Counselling practice time due to covid-19	38(21.1)	29(16.1)	63(35)	50(27.8)
Shortage of personal protective equipment	28(15.6)	34(18.9)	74(41.1)	44(24.4)

### Potential barriers of medication counseling practice

Study participants claimed that lack of knowledge (33.9%), lack of updated drug information (39.3%), high patient load (69.7%) and absence of private counselling room (55.4%) were the main factors that prohibit pharmacists from counselling their patients. About 23% of pharmacists underestimated the benefits of counselling, while 9% of them considered medication counselling were not their professional duty (Table 4).

**Table 4:** Potential barriers of medication counseling practice for pharmacists in drug retail outlets of Jimma town, Southwest Ethiopia, 2021 (n=180)

Counseling barriers	Strongly Agree (%)	Agree (%)	Neutral (%)	Disagree (%)	Strongly Disagree (%)
Lack of knowledge	35(19.4)	37(20.6)	25(13.9)	42(23.3)	41(22.8)
Limited access to updated drug information	27(15)	41(22)	34(18.9)	48(26.7)	30(16.7)
High patient load	61(33.9)	40(22.2)	38(21.1)	26(14.4)	15(3.8)
Absence of private counselling room	61(33.9)	24(13.3)	36(20)	34(18.9)	25(13.9)
Inability to communicate with the patient	24(13.3)	45(25)	12(6.7)	77(42.8)	22(12.2)
Lack of confidence	19(10.6)	46(25.6)	19(10.6)	60(33.3)	36(20)
Lack of interest	3(1.7)	44(24.4)	21(11.7)	70(38.9)	42(23.3)
Lack of experience	18(10)	52(28.9)	12(6.7)	50(27.8)	48(26.7)
Underestimation of the benefit of counselling	24(13.3)	17(9.4)	26(14.4)	42(23.3)	71(39.4)

### Perceived level of medication counseling

Ninety-two (51.1%) of the pharmacists perceived they were provided good medication counseling for their patients, while around half of the participants reported they offered inadequate medication counseling (Table 2).

### Factors associated with pharmacists counselling practice

A total of 15 variables were candidates for multivariable regression at a p-value of 0.25 on bivariate analysis. In multivariable logistic regression, pharmacists who were female, diploma level and working in public sector were less likely offered adequate patient's medication counseling 99.72% (AOR=0.0028; CI: 95%, 0.002–0.245; p=0.001), 93.9% (AOR=0.061; CI: 0.009–0.433; p= 0.005) and 90.6% (AOR=0.094; CI:0.013–0.663; p=0.018), respectively. Similarly, low pharmacist's communication level with the patients, shortage of personal protective equipment and time amid covid-19 were 99.2% (AOR=0.008; CI: 0.001–0.292; p= 0.009), 99.7% (AOR=0.003; CI: 0.001–0.114; p=0.002) and 97.9% (AOR: 0.021; CI: 0.002–0.226; p= 0.021) less likely provided adequate medication counseling, respectively. In addition, pharmacists' limited access to updated drug information (AOR= 8.89; CI: 1.529–51.7; p= 0.015) and their underestimation of the benefit of counseling (AOR=0.004; CI: 0.001–0.062; p=0.001) were major factors associated with pharmacist's inadequate medication counseling (Table 5).

**Table 5:** Factors associated with pharmacist's medication counseling practice in drug retail outlet of Jimma town, South west Ethiopia, 2021 (n=180)

Variables	Category	Counseling practice		Univariate Analysis		Multivariate Analysis	
		Good(n=92)	Poor(n=88)	COR (95% CI)	P-value	AOR (95%CI)	P-value
Sex	Female	51(55.4)	27(30.7)	1			
	Male	41(44.6)	61(69.3)	0.356(0.193–0.656)	0.001	0.0028(0.003–0.245)	0.001
Age	<30 years	44(47.8)	50(56.8)	1.435(0.798–2.583)	0.001		
	≥30	48(52.2)	38(43.2)	1			
Educational level	Diploma	10(20.4)	39(79.6)	1			
	Degree and above	82(62.6)	49(37.4)	0.153(0.07–0.334)	0.001	0.061(0.009–0.433)	0.005
Working sector	Public	78(84.8)	34(38.6)	0.113(0.055–0.23)	0.001	0.094(0.013–0.663)	0.018
	Private	14(15.2)	54(61.4)	1			
Reduced the level of communication with patients amid Covid-19	No	15(16.3)	30(34.1)	1			
	Yes	77(83.7)	58(65.9)	0.377(0.186–0.764)	0.007	0.008(0.001–0.292)	0.009
Counseling of the patient is difficult amid Covid-19	No	55(59.8)	16(18.2)	1			
	Yes	37(40.2)	72(81.2)	6.689(3.377–13.249)	0.001		
Shortage of counselling practice time due to covid-19	No	52(56.5)	15(17)	6.327(3.167–12.637)	0.001	0.021(0.002–0.226)	0.021
	Yes	40(43.5)	73(83)	1			
Shortage of personal protective equipment amid Covid-19	No	43(46.7)	19(21.6)	3.187(1.66–6.119)	0.001	0.003(0.001–0.114)	0.002
	Yes	49(53.3)	69(78.4)	1			
Lack of knowledge	Yes	27(29.3)	70(79.5)	9.362(4.718–18.578)	0.001	25.8(4.44–150)	0.001
	No	65(70.7)	18(20.5)	1			
Limited access to updated drug information	Yes	38(41.3)	64(72.7)	3.789(2.026–7.089)	0.001	8.89(1.529–51.7)	0.015
	No	54(58.7)	24(27.3)	1			
High patient load	Yes	42(45.7)	59(67)	2.422(1.323–4.435)	0.004	6.72(1.093–41.337)	0.004
	No	50(54.3)	29(33)	1			
Absence of private counselling room	Yes	32(34.8)	53(60.2)	2.839(1.55–5.201)	0.001		
	No	60(65.2)	35(39.8)	1			
Lack of confidence	Yes	36(39.1)	48(54.5)	1.867(1.032–3.337)	0.039		
	No	56(60.9)	40(45.5)	1			
Lack of interest	Yes	28(30.4)	40(45.5)	1.905(1.034–3.509)	0.039		
	No	64(69.6)	48(54.5)	1			
Underestimation of the benefit of counselling	Yes	47(51.1)	20(22.7)	1			
	No	45(48.9)	68(77.3)	0.282(0.148–0.537)	0.001	0.004(0.001–0.062)	0.001

## Discussion

Patient medication counseling at a drug retail outlet is the last phase of the pharmaceutical care delivery points where health professionals should deliver clear, correct, comprehensive, and complete directions to clients on how to receipts or use drugs. In other words, failure to do this by the pharmacist results in the patients' discontinuation of the medication, drug resistance, adverse medication reactions, increased treatment costs, lower quality of life, and overall poor patient treatment outcome. Currently, medication counseling is one of the pharmaceutical care services areas mainly affected by the covid-19 pandemic as pharmacists are at the forefront of this war 2,3,21.

According to the World Health Organization medicine use indictor, the percentage of good counselling on the dispensed drugs should be 100% 4. Despite this, the current results show that only 92 (51.1%) pharmacists have reported that they have provided adequate medication counseling and, this finding is comparable to most African countries, where more than 50% of drugs are prescribed and dispensed inappropriately. Incorrect medication use is the cause of loss of resources and patient dissatisfaction 20.

In the current study, pharmacists who constantly provided medication information on the duration of therapy (81.7%), drug name and unit dose (66.7%), and the frequency of administration (57.8%) for their clients were reported. This finding is lower than the similar studies conducted in Bench Sheko Zone, southwest Ethiopia 22 (90%, 56%, 90%), Bahir Dar, Northwest Ethiopia 3 (74%, 99.2%, 96%), Tikur-Anbessa Specialized Hospital, Ethiopia 23 (90%, 92%, 92.5%), and Qassim region, Saudi Arabia 24 ( 83%, 83%, 83%) pharmacists were delivered medication information constantly on the duration of therapy, drug name and unit dose, and frequency of administration, respectively for their patients. But it is higher than the study conducted in Saudi Arabia, where only 46.8% and 6.6% of pharmacists delivered drug information on the duration of therapy and name of the drug constantly 25. The possible reasons for this discrepancy might be the result of the Covid-19 epidemic, research population, and data collection methods. The current study used self-administered questionnaires, whereas the study in Bench Sheko Zone, Bahir Dar, Tikur-Anbessa Specialized Hospital, and Saudi Arabia was conducted through the observational checklist.

Low pharmacist's level of communication with the patients due to Covid-19 (AOR=0.008; CI: 0.001–0.292; p= 0.009) were provided 99.2% less likely inadequate medication counseling. In addition to shortage of personal protective equipment (AOR=0.021; CI: 0.002–0.226; p=0.002), and shortage of time (AOR: 0.003; CI: 0.001–0.114; p= 0.002) amid covid-19 were the major factors associated with inadequate medication counselling. This finding was supported by the study conducted in Gondar and Bahir Dar towns 14, where 82.2% and 97.1% of the study participants reported they committed a shortage of personal protection equipment to deliver pharmacy services. Additionally, these studies were reported similar findings with the current study, where the level of communication and shortage of personal protection equipment were the major predictors of inadequate pharmacist medication counseling during the COVID-19 outbreak. These brought a significant impact on the pharmacist's medication counseling practice where it led to poor treatment outcomes, patient dissatisfaction, and raised unnecessary health care costs.

The pharmacist's working in public drug retail outlets, 90.6% (AOR=0.094; CI: 0.013–0.663; p=0.018), were less likely to offer adequate medication counseling than private sectors. This finding was supported by the study conducted at Bahir Dar city 3. This may be due to private drug outlets are often concerned about attracting customers to promote and improve their market value, as well as the stability of care. Pharmacists limited access to updated drug information (AOR= 8.89; CI: 1.529–51.7; p= 0.015) were associated with inadequate medication counseling in this study which supported with studies conducted in Mekele city 9, Bahir Dar city 3, Gondar, Ethiopia 26, and Tikur-Anbessa specialized hospital 23. Also, pharmacists' underestimation of the benefit of medication counseling (AOR=0.004; CI: 0.001–0.062; p=0.001) was associated with inadequate medication counseling, which was supported by the study conducted in Mekele city 9. This may be due to the pharmacist's tendency to traditional perception and the lack of structured teaching sections so that professionals can motivate and raise awareness about practicing medication counseling. The pharmacist's underestimation of medication counseling and lack of understanding are the risk factors for inadequate medication counseling. The patient' non adherence to the treatment due to pharmacists' inadequate medication counseling is associated with morbidity, mortality rates, and rising health costs 27.

Pharmacists who were working at drug retail outlets with high patient loads were 6.72 times less likely to provide adequate medication counseling (AOR=6.72; CI: 1.093–41.337; p=0.004), this is in line with studies conducted in Lagos, southwest Nigeria 28 Swedish 29, Mekele city, Northern Ethiopia 9, Bahir Dar city, Northwest Ethiopia 3. Pharmacists' medication counseling with a smaller number of the patient was more likely to have better satisfaction than high patient loads.

### The current study has limitations

The study data collection was conducted through self-administered questioners. Due to covid -19 and financial issue we didn't conduct observation study. Lack of similar study conducted amid covid-19 for comparisons is another challenge.

## Conclusion

About half of the pharmacists reported that they were inadequately delivered medication counseling for their patients in the current study. Reduced pharmacist's level of communication with the patients and shortage of personal protective equipment due to Covid-19, patient load, pharmacists limited access to updated drug information, underestimation of the benefit of counseling, and working in public drug retail outlets were major factors associated with medication counseling practice. In general, Jimma town health offices and Oromia Region Health bureau should effort in association with other stakeholders to improve the identified bottleneck of pharmacist's medication counselling practice.

## Data Availability

The datasets are available from the corresponding author upon reasonable request

## Abbreviations

AOR: Adjust odd ratio
COVID-19: Coronavirus disease 2019
ETB: Ethiopian Birr
SPSS: Statistical Package for the Social Sciences
WHO: World Health Organization
